# Antitumour activity of pembrolizumab in advanced mucosal melanoma: a post-hoc analysis of KEYNOTE-001, 002, 006

**DOI:** 10.1038/s41416-018-0207-6

**Published:** 2018-09-11

**Authors:** Omid Hamid, Caroline Robert, Antoni Ribas, F. Stephen Hodi, Euan Walpole, Adil Daud, Ana S. Arance, Ewan Brown, Christoph Hoeller, Laurent Mortier, Jacob Schachter, Jianmin Long, Scot Ebbinghaus, Nageatte Ibrahim, Marcus Butler

**Affiliations:** 1 The Angeles Clinic and Research Institute, Los Angeles, CA USA; 2 Gustave Roussy and Paris-Sud University, Villejuif, France; 3 University of California, Los Angeles, Los Angeles, CA USA; 4 Dana-Farber Cancer Institute, Boston, MA USA; 5 Princess Alexandra Hospital, Brisbane, Australia; 6 University of California, San Francisco, San Francisco, CA USA; 7 Hospital Clinic de Barcelona, Barcelona, Spain; 8 Edinburgh Cancer Research Centre and Western General Hospital, Edinburgh, Scotland UK; 9 Medical University of Vienna, Vienna, Austria; 10 Centre Hospitalier Régional Universitaire de Lille, Université Lille, Lille, France; 11 Ella Lemelbaum Institute for Melanoma, Sheba Medical Center, Tel Hashomer, Israel; 12 Merck & Co., Inc, Kenilworth, NJ USA; 13 Princess Margaret Cancer Centre, Toronto, ON Canada

**Keywords:** Melanoma, Skin cancer

## Abstract

**Background:**

Mucosal melanoma is an aggressive melanoma with poor prognosis. We assessed efficacy of pembrolizumab in patients with advanced mucosal melanoma in KEYNOTE-001 (NCT01295827), −002 (NCT01704287), and −006 (NCT01866319).

**Methods:**

Patients received pembrolizumab 2 mg/kg every 3 weeks (Q3W) or 10 mg/kg Q2W or Q3W. Response was assessed by independent central review per RECIST v1.1.

**Results:**

1567 patients were treated and 84 (5%) had mucosal melanoma. Fifty-one of 84 were ipilimumab-naive. In patients with mucosal melanoma, the objective response rate (ORR) was 19% (95% CI 11–29%), with median duration of response (DOR) of 27.6 months (range 1.1 + to 27.6). Median progression-free survival (PFS) was 2.8 months (95% CI 2.7–2.8), with median overall survival (OS) of 11.3 months (7.7–16.6). ORR was 22% (95% CI 11–35%) and 15% (95% CI 5–32%) in ipilimumab-naive and ipilimumab-treated patients.

**Conclusion:**

Pembrolizumab provides durable antitumour activity in patients with advanced mucosal melanoma regardless of prior ipilimumab.

## Introduction

Mucosal melanomas, a rare but aggressive subtype, represent ~1.3% of all melanoma diagnoses.^1,2^ Most occur in the head and neck (50% of cases), anorectal (25%), and vulvovaginal (20%) regions, and are more frequent in women aged 70–79 years.^2^ As there are no specific treatment guidelines for patients with advanced mucosal melanoma, therapies are the same as for advanced nonmucosal melanoma. Historically, outcomes with mucosal melanoma are poorer than cutaneous melanoma. This has been attributed to initial presentation at a later stage, with an estimated 5-year survival rate across early stage of 81%.^3^ In patients with metastatic mucosal melanoma, the 5-year survival rate is ~16%.^4^

Recent data suggest that anti-PD-1 inhibitor therapies may have antitumour activity against mucosal melanoma. In a phase 1 study of pembrolizumab in Japanese patients with advanced melanoma, ORR was 25% and 24% for 8 patients with mucosal and 29 with nonmucosal melanoma.^5^ In pooled analyses of nivolumab and ipilimumab in 889 patients with advanced melanoma (86 with mucosal melanoma), ORRs were 23 and 41% in patients with mucosal and nonmucosal melanoma who received nivolumab monotherapy, 37 and 60% in patients who received nivolumab and ipilimumab combination therapy, and 8 and 21%, respectively, for patients who received ipilimumab monotherapy.^6^

The monoclonal anti-PD-1 antibody pembrolizumab has demonstrated robust and durable antitumour activity, with a manageable safety profile in patients with ipilimumab-treated and ipilimumab-naive advanced melanoma. Here, we report the results of a post-hoc analysis assessing the efficacy of pembrolizumab in ipilimumab-naïve and ipilimumab-treated patients with mucosal melanoma enrolled in the KEYNOTE-001, 002, and 006 clinical studies.^7–9^

## Materials and methods

### Patients

Eligibility criteria were previously reported.^7–9^ Common criteria included age ≥18 years, histologically or cytologically confirmed, unresectable stage III or IV melanoma, measurable disease per Response Evaluation Criteria in Solid Tumour (RECIST) v1.1, Eastern Cooperative Oncology Group performance status 0–1, and adequate organ function. Patients receiving ≥ 1 dose of pembrolizumab are evaluated. Protocols for the studies were approved by institutional review boards at each site. All patients provided written informed consent.

### Treatment

In this exploratory, post-hoc analysis, data were pooled from three studies of patients receiving pembrolizumab until disease progression (PD) or unacceptable toxicity. KEYNOTE-001 was an open-label, multicohort, phase 1b study of pembrolizumab 2 mg/kg Q3W, 10 mg/kg Q3W, or 10 mg/kg Q2W in adults with ipilimumab-(PD-1 and PD-L1 inhibitor)-naive or treated, advanced melanoma (*N* = 655); KEYNOTE-002 was an open-label, randomised, phase 2 study of pembrolizumab 2 mg/kg or 10 mg/kg Q3W versus chemotherapy in adults with ipilimumab-refractory, advanced melanoma (*N* = 540), and KEYNOTE-006 was an open-label, randomised, phase 3 study of pembrolizumab 10 mg/kg Q3W or Q2W in ipilimumab-naive, advanced melanoma (*N* = 834). Investigators were not required to indicate the location of the primary mucosal melanoma lesion.

### Efficacy assessments and statistical considerations

Response was assessed per RECIST v1.1 by independent central review Q12W in KEYNOTE-001, at week 12 and every 6 weeks through week 48, then Q12W thereafter in KEYNOTE-002 and KEYNOTE-006. Survival was assessed Q12W in all studies. Objectives included summarising baseline and disease characteristics, evaluating ORR (complete response (CR) + partial response (PR)), disease control rate (DCR (CR + PR + stable disease (SD))), duration of response (DOR): time from CR or PR to first PD, progression-free survival (PFS): time from treatment start to first PD or death, and overall survival (OS): time from treatment start to death from any cause, in patients with advanced mucosal melanoma, and assessment of ORR, DCR, PFS, and OS in patients with mucosal and nonmucosal melanoma. PFS, OS, and DOR were estimated using the Kaplan–Meier method.

## Results

### Baseline characteristics

Eighty-four of 1567 (5%) patients receiving ≥ 1 dose of pembrolizumab had mucosal melanoma (Table 1); 36 of 655 (5%) in KEYNOTE-001, 11 of 357 (3%) in KEYNOTE-002, and 37 of 555 (7%) in KEYNOTE-006. Baseline characteristics between patients with mucosal and nonmucosal melanoma were comparable including age, ECOG performance status, stage M1c disease, presence of liver metastases and prior ipilimumab. A larger proportion of patients with mucosal melanoma had ≥ 2 prior therapies and were PD-L1 negative (Table 1). Characteristics with ≥ 10% difference between the two groups included female sex (57% and 38%; *P* = 0.0006), elevated LDH (48% and 36%; *P* = 0.0349), overall baseline median tumour size ≥ 80.5 mm (58% and 43%; *P* = 0.0077), and *BRAF*^V600^ mutation (8 and 29%; *P* < 0.0001).

**Table 1:** Baseline disease and patient characteristics

Characteristics, *n* (%)	Mucosal *N* = 84	Nonmucosal *N* = 1483	*P*-value^c^
Age, median (range), years	64 (15–87)	61 (18–94)	0.4805
≥65 years	41 (49%)	620 (42%)	0.2061
Women	48 (57%)	568 (38%)	0.0006
ECOG PS 1	27 (32%)	513 (35%)	0.6459
Elevated LDH	40 (48%)	537 (36%)	0.0349
*BRAF*^V600^ mutant	7 (8%)	427 (29%)	<0.0001
M1c disease	68 (81%)	1102 (74%)	0.1732
Liver metastases	20 (24%)	286 (19%)	0.3089
Baseline tumour size ≥ 80.5 mm^a^	49 (58%)	645 (43%)	0.0077
PD-L1 positive^b^	46 (70%)	888 (77%)	0.1675
No. of prior systemic therapies			
0	8 (10%)	150 (10%)	0.0462
1	31 (37%)	639 (43%)	─
2	38 (45%)	467 (31%)	─
≥3	7 (8%)	227 (15%)	─
Prior chemotherapy	18 (21%)	233 (16%)	0.1646
Prior ipilimumab	33 (39%)	666 (45%)	0.3131

*ECOG* eastern cooperative oncology group, *LDH* lactate dehydrogenase

^a^Baseline tumour size is the sum of the longest diameters of target lesion. 80.5 mm is the median in the total population

^b^Percentage is calculated using the number of patients with known PD-L1 status as the denominator (*n* = 66 for mucosal and 1152 for nonmucosal)

^c^Based on *t*-test for age and on chi-square test for other characteristics

### Efficacy

**Fig. 1:**
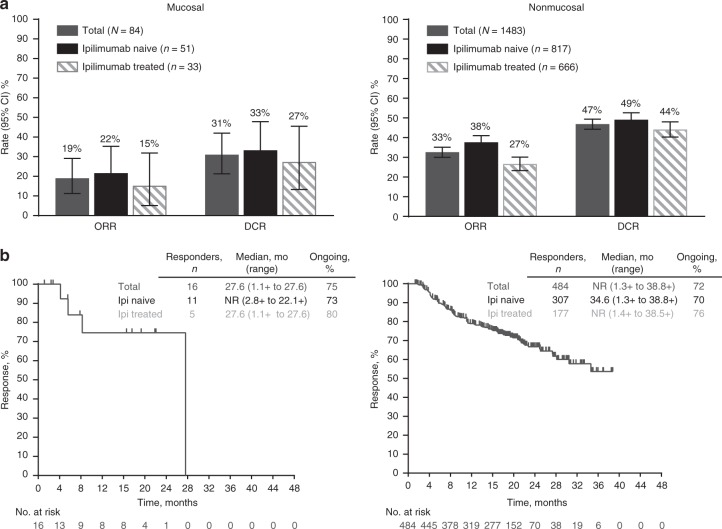
Antitumour activity. Response rates (**a**) and durability of response (**b**) with pembrolizumab in mucosal and nonmucosal melanoma

In patients with mucosal melanoma, ORR was 19% (95% CI 11–29%) overall, 22% (95% CI 11–35%) in ipilimumab-naive, and 15% (95% CI 5–32%) in ipilimumab-treated patients (Fig. 1a). ORRs were 13% (1 of 8) and 20% (15 of 76) in patients with zero and ≥1 prior therapy, respectively. In nonmucosal melanoma, ORR was 33% (95% CI 30–35%) overall, 38% (95% CI 34–41%) in ipilimumab-naive, and 27% (95% CI 23–30%) in ipilimumab-treated patients. ORRs were 42% (63 of 150) and 32% (421 of 1333) in patients with zero and ≥1 prior therapy, respectively. The DCR was 31% (95% CI 21–42%) with 19% CR + PR and 12% SD for patients with mucosal and 47% (95% CI 44–49%) with 33% CR + PR and 14% SD for those with nonmucosal melanoma (Fig. 1a).

For the 16 responders with mucosal melanoma, median time to response was 2.8 months (range 2.6–19.4), and median DOR was 27.6 months (range 1.1 + months to 27.6 months) overall, with 75% responses ongoing at the time of data cutoff (Fig. 1b). For ipilimumab-naive patients (*n* = 11), median time to response was 2.8 months (2.8–16.8) and median DOR was not reached (range 2.8 + to 22.1 + months) with 73% of responses ongoing. For ipilimumab-treated patients (*n* = 5), median time to response was 4.4 months (2.6–19.4) and median DOR was 27.6 months (range 1.1 + to 27.6) with 80% of responses ongoing (Fig. 1b). In the 484 responders with nonmucosal melanoma, median DOR was not reached (range 1.3 + to 38.8 + months), with 72% of responses ongoing (Fig. 1b). Median DOR was 34.6 months (range 1.3 + to 38.8 + ) for ipilimumab-naive patients (*n* = 307), with 70% of responses ongoing, and was not reached (range 1.4 + to 38.5 + months) for ipilimumab-treated patients (*n* = 177), with 76% of responses ongoing.

**Fig. 2:**
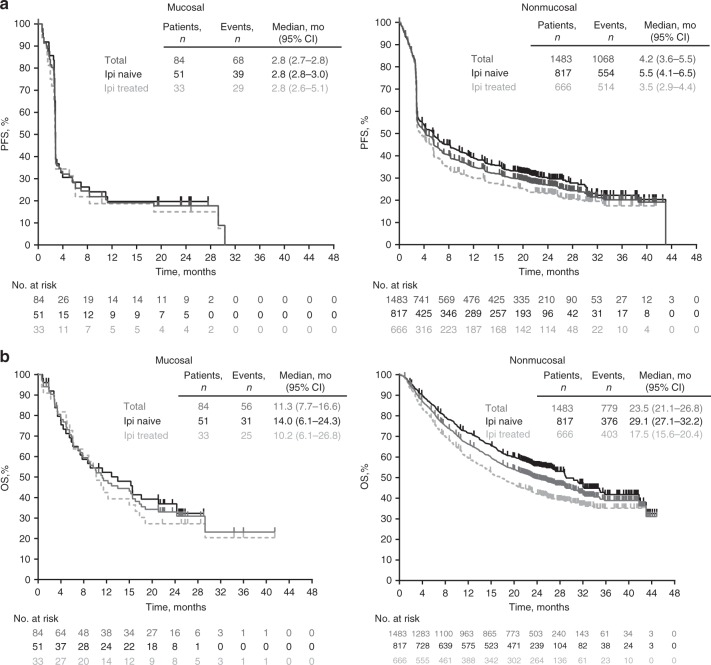
Survival outcomes. Progression-free (**a**) and overall survival (**b**) with pembrolizumab in mucosal and nonmucosal melanoma

Median PFS was 2.8 months (95% CI 2.7–2.8) overall in patients with mucosal melanoma, and 2.8 months for both ipilimumab-naïve (2.8–3.0) and ipilimumab-treated (2.6–5.1) patients (Fig. 2a). In nonmucosal melanoma, median PFS was 4.2 months (3.6–5.5) overall, and 5.5 months (4.1–6.5) and 3.5 months (2.9–4.4) for ipilimumab-naive and ipilimumab-treated patients (Fig. 2a). Median OS was 11.3 months (95% CI 7.7–16.6) overall in patients with mucosal melanoma, and 14.0 months (6.1–24.3) and 10.2 months (6.1–17.1), respectively, for ipilimumab-naive and ipilimumab-treated patients. In nonmucosal melanoma, median OS was 23.5 months (21.1–26.8) overall, and 29.1 months (27.1–32.2) and 17.5 months (15.6–20.4), respectively, in ipilimumab-naive and ipilimumab-treated patients (Fig. 2b).

### Safety

Sixty-one of 84 (73%) patients with mucosal melanoma and 1203 of 1483 (81%) with nonmucosal melanoma had a treatment-related adverse event (AE). Most treatment-related AEs were low grade. Eight (10%) patients with mucosal melanoma and 263 (18%) with nonmucosal melanoma had a grade 3–4 treatment-related AE, and 3 (0.2%) patients with nonmucosal melanoma had a grade 5 treatment-related AE of general physical deterioration, sepsis, and respiratory failure in one patient each (Supplementary Table).

## Discussion

This post-hoc analysis showed that pembrolizumab provided durable antitumour activity with clinically relevant benefit in patients with advanced mucosal melanoma regardless of prior ipilimumab. The ORR was 19% and median time to response was 2.8 months for patients with mucosal melanoma who received pembrolizumab. Responses were similar among ipilimumab-naïve and ipilimumab-treated patients. Consistent with previous reports, responses were lower in patients with mucosal versus nonmucosal melanoma (ORR 19% versus 33%), but appeared similarly durable with 75% and 72% of patients, respectively, having an ongoing response without progression. Although survival seemed shorter in patients with mucosal versus nonmucosal melanoma (median PFS of 2.8 months versus 4.2 months and median OS of 11.3 months versus 23.5 months), the benefit in patients with mucosal melanoma appeared clinically relevant. However, longer follow-up will be needed to confirm that durable responses translate into a higher proportion of patients achieving long-term survival benefit.

Mucosal melanomas have strikingly different biologic and molecular profiles compared with nonmucosal melanomas that, in addition to differences in originating anatomic location, may contribute to lower efficacy outcomes.^10,11^ In this study, a higher proportion of patients with mucosal melanoma had elevated LDH levels and overall baseline median tumour size ≥80.5 mm, and fewer had BRAF^V600^ mutation compared to patients with nonmucosal melanoma, all statistically significant differences. In addition, ~45% and 31% of patients with mucosal and nonmucosal melanoma had ≥2 prior therapies and 30% and 23%, respectively, were PD-L1 negative, characteristics associated with lower efficacy in patients with advanced melanoma.^9^ However, comparison of outcomes between mucosal and nonmucosal melanoma should be interpreted with caution given the post-hoc nature of this analysis, differences in baseline characteristics, and the small number of patients with mucosal melanoma. Lack of anatomic information is an additional limitation. Going forward, combination regimens may be of greater benefit in mucosal melanoma.^6^ Ongoing studies of PD-1 inhibitors in mucosal melanoma include nivolumab and ipilimumab (NCT02978443), and pembrolizumab and epacadostat (NCT02752074). In summary, as in nonmucosal melanoma, pembrolizumab provided a durable antitumour benefit for responding patients and is an effective treatment for advanced mucosal melanoma.

## Electronic supplementary material

Supplementary Table

## Availability of data and material

Merck Sharp & Dohme's (a subsidiary of Merck & Co., Inc., Kenilworth, NJ) data sharing policy, including restrictions, is available at http://engagezone.merck.com/ds_documentation.php. Requests for access to the clinical study data can be submitted through the EngageZone site or via email to dataaccess@merck.com.
