# Supplementary material for: Antitumour activity of pembrolizumab in advanced mucosal melanoma: a post-hoc analysis of KEYNOTE-001, 002, 006
Source: Br J Cancer. 2018 Sep 11;119(6):670–4. doi: 10.1038/s41416-018-0207-6 (PMC6173747; doi:10.1038/s41416-018-0207-6)
Supplement: Supplementary file 1 — Supplementary Table [file 41416_2018_207_MOESM1_ESM.docx]

| **Table S1. Treatment-related adverse events** | | |
| --- | --- | --- |
| **Events, n (%)** | **Mucosal**  **N = 84** | **Nonmucosal**  **N = 1483** |
| Any | 61 (73%) | 1203 (81%) |
| Grade 3-5 | 8 (10) | 266 (18) |
| **Observed in ≥10% of patients in any treatment group** | | |
| Pruritus | 22 (26) | 357 (24) |
| Fatigue | 17 (20) | 490 (33) |
| Nausea | 15 (18) | 192 (13) |
| Vitiligo | 13 (16) | 172 (12) |
| Diarrhea | 12 (14) | 267 (18) |
| Arthralgia | 11 (13) | 214 (14) |
| Decreased appetite | 11 (13) | 121 (8) |
| Rash | 8 (10) | 281 (19) |
| Asthenia | 8 (10) | 147 (10) |
